# Comparison of magnetic resonance imaging sequences for depicting the subthalamic nucleus for deep brain stimulation

**DOI:** 10.1007/s12194-014-0283-0

**Published:** 2014-08-12

**Authors:** Hiroshi Nagahama, Kengo Suzuki, Takaharu Shonai, Kazuki Aratani, Yuuki Sakurai, Manami Nakamura, Motomichi Sakata

**Affiliations:** 1 Department of Radiology and Nuclear Medicine, Sapporo Medical University Hospital, Chuo-ku, Minami-1, West-16, Sapporo, 060-8543 Japan; 2 Department of Neurosurgery, Sapporo Medical University Hospital, Chuo-ku, Minami-1, West-16, Sapporo, 060-8543 Japan; 3 Department of Radiology, Sapporo Medical University Hospital, Chuo-ku, Minami-1, West-16, Sapporo, 060-8543 Japan; 4 Graduate School of Healthcare Sciences, Hokkaido University, Kita-ku, Kita-12, West-5, Sapporo, 060-0812 Japan

**Keywords:** Deep brain stimulation, Subthalamic nucleus, Brain iron, Susceptibility, Imaging sequence

## Abstract

Electrodes are surgically implanted into the subthalamic nucleus (STN) of Parkinson’s disease patients to provide deep brain stimulation. For ensuring correct positioning, the anatomic location of the STN must be determined preoperatively. Magnetic resonance imaging has been used for pinpointing the location of the STN. To identify the optimal imaging sequence for identifying the STN, we compared images produced with T_2_ star-weighted angiography (SWAN), gradient echo T_2_*-weighted imaging, and fast spin echo T_2_-weighted imaging in 6 healthy volunteers. Our comparison involved measurement of the contrast-to-noise ratio (CNR) for the STN and substantia nigra and a radiologist’s interpretations of the images. Of the sequences examined, the CNR and qualitative scores were significantly higher on SWAN images than on other images (*p* < 0.01) for STN visualization. Kappa value (0.74) on SWAN images was the highest in three sequences for visualizing the STN. SWAN is the sequence best suited for identifying the STN at the present time.

## Introduction

Deep brain stimulation (DBS) of the subthalamic nucleus (STN) via electrical stimulation with implanted electrodes improves the quality of life of Parkinson’s disease patients [1, 2]. The fine structure of the STN, which is only several millimeters in diameter, requires highly accurate localization to allow proper implantation of the electrodes, which are about 1.5 mm in height [3, 4]. Investigations with T_2_-weighted spin echo magnetic resonance imaging (MRI) used as a method for direct identification of the STN location have occasionally been reported [5, 6]. The iron content of the STN affects MRI rendering of the STN [6, 7]. Gradient echo (GRE) T_2_*-weighted imaging and T_2_ star-weighted angiography (SWAN) are both highly sensitive to differences in magnetic susceptibility, and they may thus be able more clearly to render the STN. We compared images produced with 3 sequences—SWAN, GRE-T_2_*-weighted imaging (GRE-T_2_*), and fast spin echo (SE) T_2_-weighted imaging (FSE-T_2_)—to identify the sequence best suited for determining the STN location.

## Methods

### Equipment and subjects

A 3.0-T MRI scanner (SignaHDxt; GE Healthcare, Milwaukee, WI, USA) with an 8-channel phased array coil was used for head imaging. SPSS Statistics version 20 software (IBM, Armonk, NY, USA) was used for statistical analyses. OsiriX software (version 4.1.2, Pixmeo, Geneva, Switzerland) was used for image analysis. An iMac (Mid-2011 model; Apple Computer, Cupertino, CA) was used for visual assessment of the STN. Subjects were 6 healthy volunteers [4 men, 2 women; mean age, 34.3 ± 8.3 (standard deviation) years; age range 26–51 years]. The study was conducted with the approval of the ethics committee at our medical institution. The objective and contents of the experiment were fully explained to the volunteers before informed consent was obtained.

### Imaging sequences

The imaging conditions for the sequences used were as follows: SWAN was performed with 3-dimensional (3D) imaging with: repetition time (TR)/echo time (TE) = 86.8/46.7 (13.0, 24.2, 35.4, 46.7, 57.9, 69.2, 80.4) ms, flip angle (FA) = 20°, bandwidth (BW) = ±31.2 kHz, field of view (FOV) = 256 mm, slice thickness (Thk)/spacing (Sp) = 1.0/0.0 mm, matrix = 384 × 384 (pixel size = 0.67 mm), number of signal averages (NSA) = 1, total number of slices = 52, acquisition time (AT) = 8:24, and parallel imaging (phase reduction factor = 2).

GRE-T_2_* was performed with 2-dimensional (2D) imaging with: TR/TE = 680/25 ms, FA = 30°; BW = ±15.6 kHz, FOV = 180 mm, Thk/Sp = 2.0/0.5 mm, matrix = 352 × 352 (pixel size = 0.51 mm), NSA = 2, total number of slices = 15, and AT = 8:03.

Finally, FSE-T_2_ was performed with 2D imaging with: TR/TE = 4000/80 ms, FA = 90°, BW = ±50 kHz, FOV = 180 mm, Thk/Sp = 2.0/0.5 mm; matrix = 352 × 352 (pixel size = 0.51 mm), NSA = 8, total number of slices = 15, and AT = 8:08. The aim of this study was to select the best sequence for clinical use. Because the imaging quality varied with the imaging parameters (imaging sequence, TR, TE, 2D or 3D, etc.), the imaging times of the sequences were kept as close to each other as possible.

### Imaging methods

**Fig. 1:**
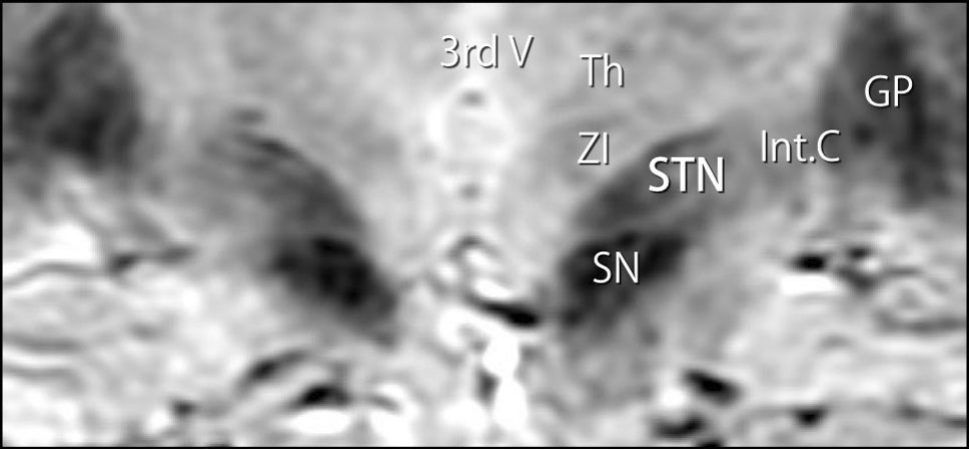
Anatomy of the STN and surrounding features as imaged with SWAN. *GP* globus pallidus, *Int. C* internal capsule, *Th* thalamus, *SN* substantia nigra, *STN* subthalamic nucleus, *ZI* zonaincerta, *3rd V* third ventricle

Coronal imaging was used. The slices were set for left–right symmetry of the basal ganglia based on a T_2_-weighted cross-sectional image connecting the anterior and posterior commissures. The range of imaging was set so that the dorsal sides included the cerebral aqueduct and the ventral sides included the anterior commissure. The anatomy of the STN and surrounding features in coronal images produced with these settings is shown in Fig. 1.

### Image analysis

#### Contrast-to-noise ratio (CNR) analysis

**Fig. 2:**
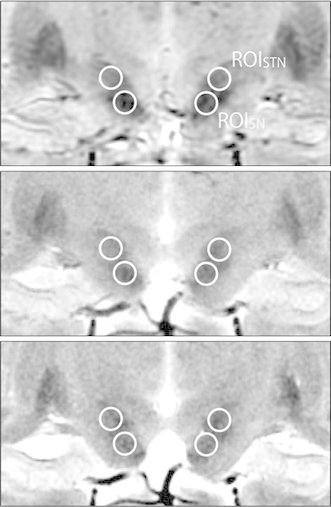
Coronal image by SWAN (*top*), GRE-T_2_* (*middle*), and FSE-T_2_ (*bottom*). *Circles* in the figure represent ROIs for CNR assessment. ROI_STN_, region of interest to STN; ROI_SN_, region of interest to SN

A circular 0.12 cm^2^ region of interest (ROI) that included at least 50 pixels was placed in the left and right STN and the substantia nigra (SN) in identical slices of any one of the three sequences, and the ROIs were propagated onto the other two sequences (Fig. 2) [8–11]. The signal intensity in the ROIs was measured by a radiologic technologist with 8 years experience three times per day on both sides, and the mean was used for determination of the CNR according to the following formula:

$${\text{CNR}} = \left( {{\text{SI}}_{\text{STN}} - \; {\text{SI}}_{\text{SN}} } \right)/{\text{SD}}_{\text{STN}} ,$$

where SI_STN_ is the mean signal intensity of the STN ROI, SI_SN_ is the mean signal intensity of the SN ROI, and SD_STN_ is the standard deviation of the signal intensity of the STN ROI.

CNR values were determined as described for each subject, and mean values were calculated and used for comparison of the sequences. We used paired *t* tests to test for significant differences between sequences, with values of *p* < 0.01 considered to indicate significance.

#### STN visual assessment and area measurement

Two radiologists with 12 and 18 years of experience in image interpretation were asked visually to assess the STN rendering by each sequence. The assessors evaluated renderings on a 4-grade scale (3, excellent: the STN boundary visible, definitely clear; 2, good: the STN boundary clearly visible; 1, fair: the STN boundary poorly visible; 0, poor: not visible); then the mean scores from the assessors were compared between sequences. The agreement was evaluated by use of Cohen’s kappa coefficient. Image paging and adjustment of the window level and width were performed as required for characterization of the 3D structure in reference to two papers that describe the anatomic structure of the STN and its surroundings [6, 11]. All visual assessments were performed on the same monitor, which was set to a constant brightness, and with a constant room lighting level.

**Fig. 3:**
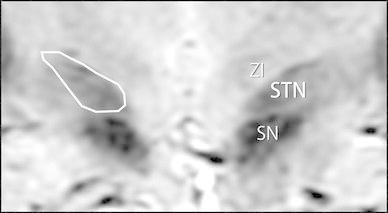
Sample of the way assessors arbitrarily drew the edges and measured the area of the STN borders on a SWAN image. *SN* substantia nigra, *STN* subthalamic nucleus, *ZI* zona incerta

Next, for quantification of the recognition of the STN, the boundaries of the STN as visually recognized by the two radiologists were arbitrary circumscribed in the specified slices of the images obtained with each sequence to allow measurement of the area of the STN (Fig. 3). The mean areas of the STN were then compared between sequences. The order of image interpretation was randomized so that the association of subjects with sequences was prevented. Left–right differences in measured areas were not compared because of the potential effects of these differences and individual differences in anatomic structure.

We used paired *t* tests to test for significant differences between sequences, with values of *p* < 0.05 considered to indicate significance.

## Results

### CNR values

**Fig. 4:**
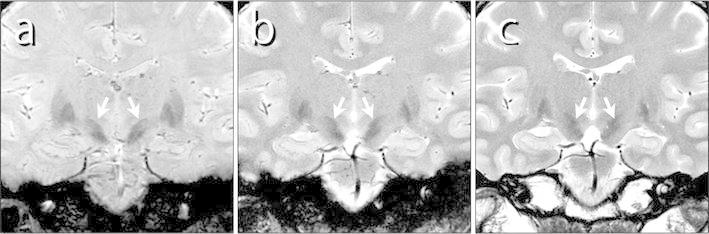
The STN (*arrows*) as imaged with the different sequences. **a** SWAN, **b** GRE-T_2_*, and **c** FSE-T_2_

**Fig. 5:**
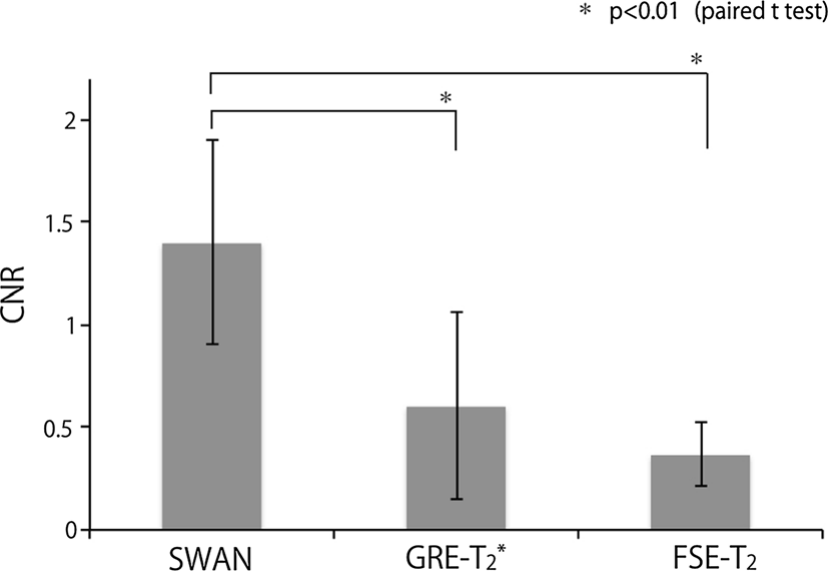
The *bar graph* shows CNR assessments. *p < 0.01, representing a significant difference from SWAN

Coronal images of the STN rendered with each sequence are shown in Fig. 4. The STN and SN appeared with lower signal intensity than those of the surrounding structures for each sequence type. The CNR for SWAN, GRE-T_2_*, and FSE-T_2_ was 1.40, 0.61, and 0.37, respectively. The CNR was significantly higher for SWAN than for FSE-T_2_ and GRE-T_2_* (*p* < 0.01) (Fig. 5). The choice of the image for setting the ROIs did not affect the CNR values.

### STN visual assessment and area measurement

**Fig. 6:**
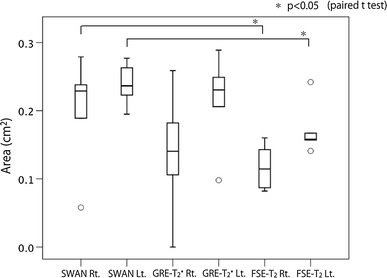
The *box plot* shows area measurements of the STN. *Left–right* differences in measured areas were not compared because of the potential effects of these differences and individual differences in anatomic structure. *p < 0.05, representing a significant difference from SWAN and FSE-T2

Assessments of the STN by the three sequences are shown in Table 1. The mean scores assigned by the two radiologists were highest for SWAN and significantly higher for SWAN than for FSE-T_2_ (*p* < 0.05). The Cohen’s kappa coefficient of 0.74 for SWAN indicated that this modality had the highest inter-assessor agreement in scores. The mean area of the portion recognized and measured as the STN was higher for SWAN than for FSE-T_2_ (Fig. 6). The mean area was significantly higher for SWAN than for FSE-T_2_ (*p* < 0.05). On the other hand, the mean area of SWAN and that of GRE-T_2_* were not statistically significantly different.

**Table 1:** Scores of agreement for the visual assessments of two radiologists

	Radiologist A	Radiologist B	Score (mean)	*K* (Cohen’s Kappa)
SWAN	2.17 ± 0.64	2.33 ± 0.75	2.25 ± 0.72^†^	0.74
GRE-T_2_*	1.50 ± 0.89	1.50 ± 0.95	1.50 ± 0.95	0.54
FSE-T_2_	1.33 ± 0.87	1.17 ± 0.69	1.25 ± 0.83	0.25

The mean scores shown indicate visual assessments of images produced with the three sequences. Cohen’s kappa coefficient indicates agreement between assessor scores

^†^ vs. FSE-T_2_ (*p* < 0.05)

## Discussion

We compared images produced with SWAN, GRE-T_2_*, and FSE-T_2_ to determine which MRI sequence is best suited for identifying the STN for DBS. Our results indicated that SWAN was the best sequence for identifying the STN for clinical use.

The coronal head images obtained in this study showed a lower signal intensity of the STN and SN than the signal intensity of the surrounding structures (Fig. 4). The difference in signal intensity in the images of each sequence is apparent visually. However, as the STN and SN both have a low signal intensity, the boundary between the two can be difficult to discern and may confound STN identification. The low signal intensity of the STN and SN in T_2_- and T_2_*-weighted imaging is attributable to the magnetic susceptibility of the iron contained in these structures [6, 12]. T_2_*-weighted imaging, with its greater sensitivity to magnetic susceptibility, therefore, produces higher contrast. However, FSE-T_2_ emits refocusing pulses in short intervals and consequently has low spin-phase dispersion and is affected minimally by magnetic susceptibility. The images produced with this sequence lack sufficient contrast to show the effects of the magnetic susceptibility of iron.

To produce images, SWAN focuses multiple echoes in a single TR, fills the k-space corresponding to each echo, and performs equilibration to form a single k-space. SWAN images thus incorporate multiple TEs. The ability of SWAN to incorporate various magnetic susceptibilities allows differences in iron deposition to be visualized as contrast [13]. The iron content of the STN varies from the ventral to the dorsal side, and lower iron deposition in the dorsal side produces less hypointensity on MRI. Iron deposition also varies with age [6, 10, 14]. The MRI contrast is thus variable. Changes in iron content must be factored in, or magnetic susceptibility must be visualized as image contrast, for proper rendering of the STN. Imaging that incorporates the contrast of multiple echoes is, therefore, useful for rendering iron-containing tissue. Realizing this, Liu et al. [9] studied the use of quantitative susceptibility mapping (QSM) for imaging of the STN.

The above findings indicate why SWAN produced a higher CNR between the STN and SN than the other sequences. SWAN is a 3D imaging sequence and, in principle, should offer a higher signal-to-noise ratio (SNR) than do the 2D sequences [15].

Finally, the multiple echoes captured include echoes with relatively high signal intensity and short TE. These echoes explain the relatively higher SNR for SWAN in comparison to GRE-T_2_* and FSE-T_2_ and contributed to a better CNR [16].

The two assessors scored SWAN images of the STN higher than those from the other sequences, with SWAN scores significantly different from FSE-T_2_ scores. These higher scores suggest that sequences highlighting magnetic susceptibility and producing 3D images may be useful for visualizing the STN. SWAN images showed high inter-assessor agreement. This finding means that the high contrast and spatial resolution of SWAN produce well-defined, recognizable boundaries. Close agreement in visual recognition among assessors facilitates preoperative planning.

**Fig. 7:**
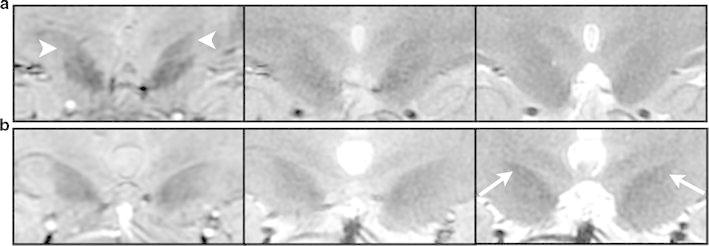
Comparison of the STN as imaged with SWAN (*left*), GRE-T_2_* (*middle*), and FSE-T_2_ (*right*). **a** The STN and SN are clearly visualized by SWAN in a 26-year-old woman (*arrowheads*), but are unclear for GRE-T_2_* and FSE-T_2_. **b** SWAN and GRE-T_2_* render the STN and SN with comparable signal intensities and with unclear boundaries in a 51-year-old man. In the FSE-T_2_ image, the STN appears with lower signal intensity than the SN (*arrow*), and the boundary between the two is clear

As the images of the healthy volunteers in Fig. 7a show, SWAN clearly depicted the STN and SN. The low contrast of the GRE-T_2_* and FSE-T_2_ images, however, resulted in poorly demarcated boundaries. The sequence used for imaging of the STN is, therefore, highly relevant, affecting the visual recognition of the STN anatomy. SWAN features a 1 mm slice thickness in comparison to the 2 mm slice thickness for the other two sequences, making the partial volume effect relevant to characterizing of the anatomic structure. The ability of SWAN to image with slices thinner than those of the other two sequences even though the three sequences have similar imaging times allows a high SNR, with 3D images produced and with multi-echo acquisition [16]. This ability was also available in our study.

As seen in Fig. 7b, SWAN sometimes produced images with the STN and SN having comparable signal intensities. In such images, the boundary separating the STN and SN was unclear. The FSE-T_2_ image in these examples showed what is presumably the STN structure with lower signal intensity than that of the surroundings, but the SN was barely visualized. In such examples, visual recognition of the STN differs significantly between SWAN and FSE-T_2_ images. Determining which image more accurately presented the STN with the images alone, however, was difficult. The representation of iron magnetic susceptibility by SWAN and GRE-T_2_* promotes a reduced signal intensity over the range of the magnetic field; therefore, image viewers must remember that the STN as imaged may appear larger than the actual anatomic structure.

Several limitations must be considered when the results of this study are interpreted. STN rendering was limited to evaluation with MRI, with no anatomic confirmation performed. The change of the imaging conditions was not considered. For instance, the TE of GRE-T_2_* was shorter than that of SWAN in this study. Because of this, for visualizing the iron-containing structure, the TE of SWAN has an advantage. When the TE is longer, GRE-T_2_* may depict the STN more clearly. However, there is some possibility that a lower SNR will be obtained. Furthermore, susceptibility-weighted imaging (SWI) and QSM technique were not available in our MRI system [9]. Therefore, we did not discuss these techniques. Other limitations related to investigation of the boundary between the STN and SN are assessment of the coronal imaging, and not considering in age-related differences for visualization of the STN. For further validation of this study, visual assessments from multi-directional views and from an elderly population without and with Parkinson’s disease are needed.

SWAN proved to be the MRI sequence most suited for identifying the STN for DBS. SWAN featured a higher CNR and a larger recognizable area than GRE-T_2_* and FSE-T_2_, was scored more highly in visual assessment, and produced images with better inter-assessor agreement.

## Conclusions

We compared images produced with SWAN, GRE-T_2_^*^, and FSE-T_2_ to determine which MRI sequence is best suited for identifying the STN for DBS. With the highest CNR, the largest area recognizable as the STN, and the best inter-assessor agreement in visual recognition, SWAN is the sequence best suited for identifying the STN at the present time.

## References

[1] Rodriguez-Oroz MC, Obeso JA, Lang AE, Houeto JL, Pollak P, Rehncrona S, Kulisevsky J, Albanese A, Volkmann J, Hariz MI (2005). Bilateral deep brain stimulation in Parkinson’s disease: a multicentre study with 4 years follow-up. Brain.

[2] Schuepbach WM, Rau J, Knudsen K, Volkmann J, Krack P, Timmermann L, Halbig TD, Hesekamp H, Navarro SM, Meier N (2013). Neurostimulation for Parkinson’s disease with early motor complications. N Engl J Med.

[3] Hamid NA, Mitchell RD, Mocroft P, Westby GW, Milner J, Pall H (2005). Targeting the subthalamic nucleus for deep brain stimulation: technical approach and fusion of pre- and postoperative MR images to define accuracy of lead placement. J Neurol Neurosurg Psychiatry.

[4] Foltynie T, Zrinzo L, Martinez-Torres I, Tripoliti E, Petersen E, Holl E, Aviles-Olmos I, Jahanshahi M, Hariz M, Limousin P (2011). MRI-guided STN DBS in Parkinson’s disease without microelectrode recording: efficacy and safety. J Neurol Neurosurg Psychiatry.

[5] Pollo C, Meuli R, Maeder P, Vingerhoets F, Ghika J, Villemure J (2003). Subthalamic nucleus deep brain stimulation for Parkinson’s disease: magnetic resonance imaging targeting using visible anatomical landmarks. Stereotact Funct Neurosurg.

[6] Dormont D, Ricciardi K, Tande D, Parain K, Menuel C, Galanaud D, Navarro S, Cornu P, Agid Y, Yelnik J (2004). Is the subthalamic nucleus hypointense on T2- weighted images? A correlation study using MR imaging and stereotactic atlas data. AJNR Am J Neuroradiol.

[7] Novellino F, Cherubini A, Chiriaco C, Morelli M, Salsone M, Arabia G, Quattrone A (2013). Brain iron deposition in essential tremor: a quantitative 3-tesla magnetic resonance imaging study. Mov Disord.

[8] Ogura A, Miyati T, Kobayashi M, Imai H, Shimizu K, Tsuchihashi T (2007). T, Machida Y. Method of SNR Determination Using Clinical Images (in Japanese). Japanese J Radiol Technol.

[9] Liu T, Eskreis-Winkler S, Schweizer A, Chen W, Kaplitt M, Tsiouris A, Wang Y (2013). Improved subthalamic nucleus depiction with quantitative susceptibility Mapping. Radiology.

[10] Kitajima M, Korogi Y, Kakeda S, Moriya J, Ohnari N, Sato T, Hayashida Y, Hirai T, Okuda T, Yamashita Y (2008). Human subthalamic nucleus: evaluation with high-resolution MR imaging at 3.0 T. Neuroradiology.

[11] Kerl HU, Gerigk L, Pechlivanis I, Al-Zghloul M, Groden C, Nolte IS (2012). The subthalamic nucleus at 7.0 tesla: evaluation of sequence and orientation for deep-brain stimulation. Acta Neurochir.

[12] Massey LA, Miranda MA, Zrinzo L, Al-Helli O, Parkes HG, Thornton JS, So PW, White MJ, Mancini L, Strand C (2012). High resolution MR anatomy of the subthalamic nucleus: imaging at 9.4 T with histological validation. Neuro Image.

[13] Du YP, Jin Z, Hu Y, Tanabe J (2009). Multi-echo acquisition of MR angiography and venography of the brain at 3 tesla. J Magn Reson Imaging.

[14] Keuken MC, Bazin PL, Schafer A, Neumann J, Turner R, Forstmann BU (2013). Ultra-high 7T MRI of structural age-related changes of the subthalamic nucleus. J Neurosci.

[15] Hashemi R, Bradley W, Lisanti C (2011). MRI: the basics.

[16] Kunimatsu A, Suzuki Y, Hagiwara K, Sasaki H, Mori H, Katsura M, Ohtomo K (2012). Clinical value of 3D T2*-weighted imaging with multi-echo acquisition: comparison with conventional 2D T2*-weighted imaging and 3D phase-sensitive MR imaging. Magn Reson Med Sci.

